# Editorial: Insights in plant symbiotic interactions: 2021

**DOI:** 10.3389/fpls.2023.1129738

**Published:** 2023-02-10

**Authors:** Andrea Genre, Katharina Pawlowski, Sabine Dagmar Zimmermann, Sergio Saia

**Affiliations:** ^1^ Department of Life Sciences and Systems Biology, University of Turin, Turin, Italy; ^2^ Department of Ecology, Environment and Plant Science, Stockholm University, Stockholm, Sweden; ^3^ IPSiM, Univ Montpellier, CNRS, INRAE, Institut Agro, Montpellier, France; ^4^ Department of Veterinary Sciences, University of Pisa, Pisa, Italy; ^5^ Centre for Climate Change Impact, University of Pisa, Pisa, Italy

**Keywords:** plant symbioses, mycorrhiza, symbiotic nitrogen fixation (SNF), plant endophytes, plant growth promoting microbes

This Research Topic was launched in late 2021 in the frame of a broad initiative covering most sections of Frontiers in Plant Science. The call for papers attracted 14 original research, method, review and mini review papers by 74 authors, largely delivering our goal to provide a transversal insight into the advances in the major symbiotic plant-microbe interactions.

The contributions embrace molecular, cellular, applicative and ecological aspects of mycorrhizas, symbiotic nitrogen fixation and plant interactions with beneficial endophytes or plant growth-promoting bacteria.

## Mycorrhizal interactions

A broad interest is focused on gene regulation in mycorrhizal symbioses (Genre et al., 2020), through genomic and transcriptomic data of mycorrhizal fungi and their hosts. Moreover, novel automated image analyses now allow a breakthrough advancement in the quantification of arbuscular mycorrhizal colonization, described in a methodological article. First, Tominaga et al. analyzed and compared transcriptional changes of symbiosis-related genes in three distinct arbuscular mycorrhizal (AM) morphotypes (Arum-, Intermediate-, Paris-type) formed by distinct host plants (*Lotus japonicus*, *Daucus carota*, *Eustoma grandiflorum*), respectively. Similarities in the expression patterns for AM marker genes, such as ammonium and phosphate transporters (Boussageon et al., 2022), were found upon colonization with the AM-fungus *Rhizophagus irregularis*, but also divergent responses to the phytohormone gibberellin. These results obtained by comparative transcriptomics open further research to dissect gibberellin-mediated regulation of mycorrhiza establishment. Such transcriptional changes associated with morphological and developmental changes involved in AM formation and turnover have been shown to be linked to GRAS transcription factors, reviewed by Ho-Plágaro and Garcia-Garrido. Genome-wide expression studies in several AM models have revealed a prominent role of this GRAS gene family in AM development, nicely summarized in a scheme. However, target genes and downstream processes need further to be studied. Such a target for root colonizing fungi is proposed by Tamayo et al. at the functional level. Indeed, overexpression of a SWEET-type monosaccharide transporter in potato favored root colonization by the AM fungus *R. irregularis* and also by the pathogenic fungus *Fusarium oxysporum*, indicating that an increase in sugar transfer from the host to the fungi is involved in the fungus-plant interaction. This study showing induction of the SWEET-type sugar transporter in AM symbiosis opens a number of questions regarding regulation under natural conditions with a multitude of interactions.

Quantification of root colonization by AM fungi is often needed for such studies and so far commonly used by manual techniques. An innovative method is proposed by Muta et al. presenting in a methodological article a software-based implementation called TAIM (Tool for Analyzing root images to calculate the Infection rate of arbuscular Mycorrhizal fungi). A similar approach was recently developed as a standalone application by Evangelisti et al. (2021) called “AMFinder” for plant root analyses using deep learning-based image processing. The novel TAIM method, described here in detail, is easily accessible from an Web-based online repository, and has the potential to revolutionize the routine in many laboratories by changing a critical activity from tedious and error-prone to rapid and reliable.

Finally, the review by Authier et al. provides a more global and ecological overview about current knowledge and research gaps concerning ectomycorrhizal (ECM) networks, found in natural but even in urban ecosystems. Mycorrhizal plants are interconnected by common mycorrhizal networks (CMNs) as described for AM plants (Wipf et al., 2019). The Authors summarize and discuss ECM-based CMNs contributing significantly to nutrient exchange, carbon trade and dynamics of plant communities, finally proposing measures for landscape and urban planning for a better use of mycorrhizal ecoservices.

## Symbiotic nitrogen fixation

Four papers deal with signaling processes in symbiotic nitrogen fixation (Downie, 2014). The review by Hawkins and Oresnik focuses on the different abiotic stresses rhizobia are exposed to during the establishment of the root nodule symbiosis – acidic pH, high osmolarity, reactive oxygen species and low oxygen levels – which serve as signals; for instance, high osmolarity leads to the induction of rhizobial *nod, nif* and *fix* genes. They also discuss the effects of nodule-specific cysteine-rich peptides (NCRs and NCR-like peptides) used by some legumes, to manipulate rhizobial differentiation. Zorin et al. examine the entire NCR gene family of pea and, based on mutants and co-expression analysis, predict transcription factors involved in their regulation. Previous studies have proposed the involvement of heterotrimeric G-proteins in the symbiotic signaling in legume/rhizobia symbioses (Pingret et al., 1998). Bovin et al. examine the genes for the G beta-subunit in two legumes, providing evidence for this subunit’s role in infection and nodule development, presumably *via* cross-talk between G-protein- and PLC-mediated signaling pathways. The review of Wang et al. discusses the multiple signals that can play a role in legume nodule induction, from plant flavonoids over rhizobial Nod factors, effectors and surface polysaccharides to plant peptides, and details the roles of individual factors in different stages of the interaction.

Another major focus was on cellular aspects of root symbioses, with two papers investigating rhizobial infection in legumes. The article by Kitaeva et al. presents original research comparing microtubule organization in the cells of determinate nodules from *Glycine max*, *G. soja*, *Phaseolus vulgaris* and *L. japonicus.* The major conclusions, based on a further comparison with cytoskeleton arrangement in indeterminate nodules, outlined interesting evolutionary and developmental implications impacting symbiosome accommodation and overall efficiency in nodule infection. The mini-review by Quilbé et al. presents an update on the most recent discoveries in the molecular control of intercellular rhizobial infection, a largely unexplored - but very common - alternative to the more studied intracellular infection *via* root hairs. The review provides a few intriguing starting points to stimulate future research, such as the apparent minor role of canonical Nod-factor signaling in intercellular bacterial accommodation, more strongly controlled by cytokinin signaling, or the peculiar infection strategies deployed by individual members of the legume family.

## Other plant growth-promoting interactions

Riesco et al. identified potential genomic features involved in the interaction between *Micromonospora* and their host plants (Trujillo et al., 2015) exploring the relationship between several tens of *Micromonospora* genomes from contrasting environments with corresponding plant-related genes. Notably, they could cluster the bacterial genomes according to solely three groups dealing with ‘plant-associated’, ‘soil/rhizosphere’, and ‘marine/mangrove’ related traits and showed that representative inocula from these latter groups produced marked differences in the plant phenotypes of an inoculated *Arabidopsis thaliana* model host. These results confirm that using bacterial genomic signatures can help to select for host colonization and the plant benefit, highlighting that the common plant growth promotion markers should not be used as sole indicators to select beneficial bacteria to be used in agronomic setups or revegetation settings.

The relationship between host plants and beneficial microbes, either AM fungi or plant growth promoting bacteria (Souza et al., 2015) were explored in two elegant studies. In particular, Zhang et al. isolated several tens of actinomycetes endophytic of several medicinal plants and tested them for the antifungal activity against *F. oxysporum* f. sp. *cubense* (Foc TR4) in banana and identified a highly beneficial strain as *Streptomyces malaysiensis*. Notably, they showed that this latter *S. malaysiensis* can stimulate the banana tolerance to Foc TR4 by both stimulating the plant expression of defense-related and antioxidant enzymes, and by exuding extracellular enzymes and metabolites with plant beneficial activity. In particular, they showed that *S. malaysiensis* 8ZJF-21 extract can inhibit the germination and growth of Foc TR4 in an *in vitro* assay and identified nineteen volatile organic compounds produced by *S. malaysiensis* as potential antifungal compounds. Azizi et al. showed that inoculation of the AM fungal species *Funneliformis mosseae* or *R. irregularis* (singly or co-inoculated) and inoculation of the bacterial species *Pseudomonas fluorescens* and *P. putida* (singly or co-inoculated) improved the tolerance of common myrtle plantlets to drought effects but not their water-use efficiency. These effects were seen mostly under the dual inoculation rather than the single inoculation, with beneficial side effects on the nutrient dynamics in both the roots and leaves.

In conclusion, this Research Topic, bringing together 14 articles dealing with different plant-microbe associations, is clearly reflecting the ongoing research linked to the important topic of such beneficial interactions. Regarding the challenge to maintain and improve further plant growth and tolerance under limiting and stressful conditions, many scientific questions remain still open and will require answers in the future.

## Author contributions

All authors listed have made a substantial, direct, and intellectual contribution to the work and approved it for publication.
